# From Seizures to Behavioral Symptoms: A Case Series of Psychiatric Adverse Effects of Levetiracetam in Epilepsy Patients

**DOI:** 10.7759/cureus.96957

**Published:** 2025-11-16

**Authors:** Ravindra K Ganjikunta, Ramya Deepti Udaragudi, Pallavi Chalivendra, Umamaheswara Rao Kaveti, Ganesh K Mallaram

**Affiliations:** 1 Pharmacology, Sri Venkateswara Institute of Medical Sciences, Tirupati, IND; 2 Psychiatry, Sri Venkateswara Institute of Medical Sciences, Tirupati, IND

**Keywords:** antiepileptic drugs, behavioral adverse effects, behavioral changes, brivaracetam, epilepsy, levetiracetam, pharmacovigilance, psychiatric adverse effects, seizures

## Abstract

Levetiracetam (LEV) is the most commonly prescribed antiepileptic drug, which can be used as both adjunctive and first-line therapy, depending on the specific type of seizure. In some patients, LEV may cause behavioral adverse effects (BAEs), which range from irritability and aggression to mood disturbances and psychosis. This case series constitutes five epileptic patients managed with LEV who developed behavioral or psychiatric disturbances. It is one of the few case series reported in India. Five patients (three males and two females, aged 9-35 years) exhibited behavioral disturbances such as irritability, aggression, anxiety, depression, hyperactivity, and psychosis, predominantly following LEV initiation or dose escalation. These patients also have contributing factors such as a history of psychiatric illness, developmental delay, and underlying structural brain abnormalities. Among these, three patients’ behavioral symptoms recovered following dose reduction or discontinuation of LEV, and the remaining two patients’ outcome information was missing due to loss of follow-up. All these five cases were reported to the Pharmacovigilance Programme of India (PVPI) through the Adverse Drug Reaction Monitoring Centre (AMC) of Sri Venkateswara Institute of Medical Sciences (SVIMS), Tirupati, Andhra Pradesh, India. No cases of mortality or lasting psychiatric complications were reported. As per the World Health Organization Uppsala Monitoring Centre (WHO-UMC) criteria, the causality of the cases was one case as certain, two as probable, and two as possible. This case series highlights the clinical significance of behavioral and psychiatric adverse effects associated with LEV. Vigilant monitoring, timely recognition, and coordinated management between neurology and psychiatry are crucial for maintaining desirable seizure control while safeguarding mental well-being.

## Introduction

Levetiracetam (LEV) is an FDA-approved antiepileptic drug indicated as adjunctive therapy for myoclonic seizures, focal-onset seizures, and primary generalized tonic-clonic seizures in adults and in children aged four years and older. Although its exact mechanism of action remains unclear, evidence suggests that its anticonvulsant effect is closely related to its high binding affinity for the synaptic vesicle protein SV2A. Following oral administration, LEV is rapidly and almost completely absorbed and is primarily eliminated unchanged through the urine. In adults, treatment is typically initiated at 500-1,000 mg per day, with titration up to a maximum dose of 3,000 mg daily. The most common adverse effects reported include somnolence, asthenia, ataxia, and dizziness. Although less frequent, behavioral and mood disturbances represent clinically significant adverse reactions [1].

Epilepsy is among the most common chronic neurological disorders, affecting an estimated 50-70 million people worldwide [2-4]. In addition to the recurrent seizures that define the condition, individuals often experience considerable psychosocial difficulties, particularly due to associated behavioral comorbidities [3,4]. Effective management, therefore, requires not only achieving seizure control but also minimizing neuropsychiatric adverse effects related to treatment. The behavioral adverse effects (BAEs) associated with LEV are particularly important, as data suggest that up to 6-20% of patients experience new or worsening psychiatric symptoms during LEV therapy [5-7]. Such events may necessitate dose adjustment or discontinuation of therapy. Risk appears to be higher among patients with pre-existing psychiatric disorders and younger age groups [8].

This case series was collected and analysed from the Adverse Drug Reaction Monitoring Centre (AMC), Sri Venkateswara Institute of Medical Sciences (SVIMS), Tirupati, Andhra Pradesh, India, after the IEC approval (Roc. No. AS/11/IEC-1857/SVIMS/2025), documents the clinical spectrum of behavioral side effects, their temporal relationship, causality by the WHO-UMC system, ‌management, and outcomes of psychiatric adverse effects in patients receiving LEV [9]. For this, the medical records of patients from September 2024 to September 2025 were studied retrospectively. It provides clinicians with information about the awareness, prevention, and importance of timely action in managing patients with BAEs during epilepsy treatment.

## Case presentation

Case 1

A 35-year-old male (Table 1) has had a history of seizure disorder since the age of one year and has been under continuous treatment. Until 2010, he was maintained on Tab. carbamazepine 300 mg (2-1-2), Tab. levetiracetam 500 mg (1-1-2), and Tab. topiramate 25 mg (4-0-4). From 2010 onwards, he started experiencing persistent low mood, lack of interest in work, and reduced concentration, which progressively worsened. In response, his treatment was modified by reducing the dose of levetiracetam to 1,500 mg/day.

**Table 1:** Comparison of the cases of levetiracetam-induced behavioral disturbances

Case	Age/Sex	Anti-epileptic drugs	Behavioral / Psychiatric Symptoms	Action taken with Levetiracetam	Treatment for Reaction	Outcome	Causality assessment by WHO-UMC
1	35/M	Tab. Carbamazepine, Tab. Levetiracetam, Tab. Topiramate	Persistent low mood, lack of interest, anxiety, dysthymia	Discontinued	Tab. Etizolam, Tab. Clonazepam + Escitalopram	Recovered	Certain
2	21/F	Tab. Carbamazepine, Tab. Levetiracetam	Fears that someone might harm her, anger outbursts, low mood, sleep disturbances, interpersonal relationship issues at home, finger-crossing behavior during stress, crying spells	Discontinued	Tab. Clonazepam + Escitalopram, Tab. Clonazepam	Recovering	Probable
3	18/M	Tab. Carbamazepine, Syp. Levetiracetam	excessive anger, physically aggressive behavior towards family members	Discontinued	Tab. Risperidone, Tab. Aripiprazole	Recovering	Probable
4	9/F	Tab. Levetiracetam, Tab. valproate	Restless, uncooperative, walking around the room	Discontinued	Tab. Clonidine, Syp. Risperidone	Lost to follow-up	Possible
5	31/F	Tab. Levetiracetam, Tab. Clobazam, Tab. Oxcarbazepine	Conversion symptoms, psychosis, behavioral arrest	Discontinued	Tab. Trihexyphenidyl, Tab. Escitalopram, Tab. Quetiapine	Lost to follow-up	Possible

During a follow-up in 2015, the Neurology Department diagnosed him with recurrent epilepsy with right temporo-parietal gliosis with depression. As depressive symptoms persisted, levetiracetam was discontinued, and he was started on Tab. phenobarbitone 60 mg (2-1-2), Tab. carbamazepine 200 mg (2-1-2), and Tab. topiramate 50 mg (1-0-2). The patient has shown improvement in depressive symptoms. In 2017, however, levetiracetam 500 mg (1-0-1) was reintroduced for epilepsy.

Later, during his follow-up visit on 12/12/2018, the Psychiatry Department diagnosed him with a mood disorder featuring dysthymia and anxiety, along with recurrent epilepsy. Consequently, levetiracetam was discontinued again, and he was prescribed Tab. etizolam (1-0-0) and a fixed-dose combination of Tab. clonazepam 0.5 mg plus escitalopram 10 mg (0-0-1). In subsequent visits after a few months, he has shown improvement. As per the psychiatrist, the behavioral disturbances were due to levetiracetam, and it was mentioned as a levetiracetam-induced behavioral disorder. On the last visit, that is, in July 2025, the patient was doing better without any behavioral disturbances.

Case 2

A 21-year-old female (Table 1) has had a seizure disorder for the past five years and was on treatment with Tab. carbamazepine and Tab. levetiracetam. She had not been pursuing studies and had stayed at home for the last three years. On 09/08/2025, she consulted with the Neurology Department with complaints of seizure-like episodes again. The episodes were characterized by a brief period of fearful sensation and confusion, followed by loss of consciousness, with tonic-clonic movements involving all four limbs. MRI brain and EEG findings were normal. A provisional diagnosis of seizure disorder vs psychogenic non-epileptic seizures (PNES) was made. Routine investigations, including ECG, hemogram, renal function tests, liver function tests, and serum electrolytes, were found to be normal.

Additionally, she expressed persistent fears that someone might harm her, feelings of being restricted at home, anger outbursts, multiple body pains, and decreased appetite. A referral was made to the Department of Psychiatry, and she was evaluated for complaints of low mood, sleep disturbances, interpersonal relationship issues at home, finger-crossing behavior during stress, and crying spells. After examination, a provisional diagnosis of conversion disorder was made. Tab. levetiracetam was discontinued due to worsening behavioral symptoms, and she was prescribed a fixed-dose combination of Tab. clonazepam 0.5 mg plus escitalopram 10 mg (0-0-1) and Tab. clonazepam 0.25 mg (1-0-0). For seizure control, Tab. carbamazepine 400 mg (1-0-1/2) and Tab. divalproex sodium 500 mg (1-0-1) were prescribed. During her follow-up visit on 02-09-2025, she was re-evaluated, and clinical improvement was observed with respect to behavioral symptoms.

Case 3

An 18-year-old male (Table 1) has a known history of seizure disorder since the age of seven years, with a history of birth asphyxia and hypoxic-ischemic encephalopathy (HIE) resulting in global developmental delay. Initially, he experienced seizures with a frequency of once per year and was managed with Tab. carbamazepine 200 mg (1-0-1) and Syp. levetiracetam 200 mg (1-0-1). In May 2017, due to increased seizure frequency, his treatment was modified to Tab. carbamazepine 200 mg (1-0-1) and Tab. clobazam 10 mg (0-0-1). A psychiatry consultation was obtained in December 2019 due to symptoms of irritability and aggression, and he was prescribed Tab. risperidone 1 mg (0-0-1) and Tab. aripiprazole 5 mg (1-0-0). As he remained seizure-free for four years, antiepileptic medications were stopped.

However, on 26/02/2025, again he had a seizure episode lasting for 10 minutes associated with tonic clonic movements of all four limbs, frothing from the mouth, tongue bite, involuntary defecation, and loss of consciousness for about 30 minutes, not associated with involuntary micturition, following which he was prescribed Tab. levetiracetam 500 mg (1-0-1). His EEG and MRI brain were normal. On examination, he was conscious and oriented, with normal speech, no nystagmus, normal tone, power 5/5 in all limbs, and no ataxia.

On 06/05/2025, he returned with complaints of behavioral disturbances, including excessive anger and physically aggressive behavior towards family members, though without suicidal ideation. After the psychiatry consultation, levetiracetam was replaced with Tab. brivaracetam 50 mg (1-0-1). As there was no improvement in anger outbursts despite this change, on 23/07/2025, brivaracetam was tapered and stopped, and the patient was started on Tab. sodium valproate 500 mg (1-0-1). Notably, improvement in behavior was observed following the initiation of sodium valproate, as reported on 04/08/2025.

Case 4

A nine-year-old female (Table 1) had a history of perinatal insult requiring oxygen support and a neonatal intensive care unit (NICU) stay for seven days. She had seizure onset on the first day of life, which remained in remission until the age of six years while on treatment with levetiracetam and valproate. On 20 March 2025, she was brought in with a recurrence of seizures. The episode was characterized by sudden tonic posturing and clonic movements of all four limbs, followed by the regaining of sensorium after about ten minutes.

On examination, the child was restless and uncooperative and was walking around the room. Based on clinical history and MRI, findings such as hypoxic insult with gliotic changes and volume loss in bilateral parieto-occipital lobes, consistent with a diagnosis of generalized tonic-clonic seizures (GTCS) secondary to hypoxic injury, with global developmental delay were made. Levetiracetam, which she had been receiving, was discontinued due to BAEs and replaced with brivaracetam at 1 mg/kg/day. By considering the risk of polycystic ovarian disease (PCOD), valproate therapy was under gradual tapering. Due to ongoing behavioral issues and hyperactivity, a psychiatric opinion was sought. A psychiatric assessment on 11 April 2025 revealed a diagnosis of intellectual disability with global developmental delay and non-goal-directed hyperactivity, along with poor eye contact and inability to sit still. Hence, the child was prescribed Tab. clonidine 100 mcg (¼ tablet at night) and Syp. risperidone 0.5 mg (0-0-1). Due to loss of follow-up with the patient, there is no update about the outcome.

Case 5

A 31-year-old female (Table 1) has had a history of seizure disorder since the age of two years, with increased frequency noted at the age of nine years, experiencing up to five attacks per day. Her seizure semiology included sudden behavioral arrest with head deviation to the right, clonic movements at the right angle of the mouth, followed by GTCS with tongue bite and frothing, suggestive of right focal seizures with secondary bilateral tonic-clonic spread. She was initially treated with Tab. levetiracetam 1 g (1-0-1), Tab. clobazam 5 mg (1-0-1), Tab. escitalopram 5 mg (1-0-0), and Tab. rejunex (multivitamin) (0-1-0).

In March 2020, she was diagnosed with PNES and was prescribed Tab. oxcarbazepine 300 mg (1-0-1), Tab. levetiracetam 1 gm (1-0-1), and Tab. clobazam 10 mg (0-0-1). Her EEG from April 2024 and MRI from 2018 were reported as normal. On 15 February 2025, she was diagnosed with conversion disorder with psychosis, due to which Levetiracetam was discontinued and replaced with Tab. brivaracetam 100 mg (1-0-1).

On examination, she was found to be conscious, coherent, hemodynamically stable, and without any focal neurological deficits. Psychiatry consultation was sought, and she was started on Tab. trihexyphenidyl 2 mg (1-0-0), Tab. escitalopram 20 mg (1-0-0), and Tab. quetiapine 25 mg (1-0-0). As the patient has been seizure-free for the past five years during her follow-ups, a plan was made by the neurologist to initiate the tapering of clobazam. Due to loss of follow-up with the patient, there is no update about the outcome of behavioral disturbances.

Causality assessment

In our case series, causality was assessed by using the WHO-UMC system. For Case 1, as dechallenge positive and rechallenge positive information was available along with plausible temporality, the causality was assessed as “certain”. For Cases 2 and 3, as only reasonable temporality, along with dechallenge positive information was available, the causality was assessed as “probable”. However, for Cases 4 and 5, as only reasonable temporality information was available, the causality was assessed as “possible”.

## Discussion

LEV is a widely used broad-spectrum antiepileptic drug known for its favorable pharmacokinetic profile and minimal drug interactions; however, it is increasingly recognized for causing BAEs in a subset of patients. The five cases presented here highlight the spectrum of such disturbances, ranging from irritability, aggression, and hyperactivity to mood changes and psychosis, emerging after initiation or dose escalation of LEV. These manifestations have been consistently reported in clinical studies, with an incidence of BAEs estimated between 10% and 20% of treated individuals, sometimes requiring discontinuation of therapy [6-10]. Predisposing factors noted in the literature, such as pre-existing psychiatric illness, structural brain lesions, developmental delay, and polytherapy, were also observed in some of our patients, suggesting that intrinsic neurobiological vulnerability amplifies LEV’s behavioral impact [11]. The exact mechanism behind these behavioral reactions remains incompletely understood. LEV’s high-affinity binding to synaptic vesicle protein 2A (SV2A) modulates neurotransmitter release, which may alter GABAergic and serotonergic transmission and thus disturb emotional regulation circuits [12]. Such disruption of neurochemical homeostasis likely forms the basis of the behavioural dysregulation observed in susceptible individuals. Importantly, many patients experience resolution of behavioral symptoms upon dose reduction or withdrawal of LEV, reinforcing the causal association; this was also seen in three of our cases [13]. Similar to our study, a study conducted in Germany showed 33 cases of aggressive behavior on LEV therapy [14]. Nonetheless, not all patients benefit equally from substitution of antiepileptics; psychiatric comorbidities may persist independently of drug exposure [15]. A study mentioned that pyridoxine supplementation can mitigate LEV-induced BAEs [16].

Confidentiality was maintained for all the study participants, and the cases were submitted to the Pharmacovigilance Programme of India (PVPI) through AMC of SVIMS. In our case series, one case was classified as certain, two cases as probable, and two others as possible in terms of causality, based on the WHO-UMC system. Although assessing causality for these retrospective cases was challenging due to the limited information available from records, we made every effort to do so. Establishing a definitive link between LEV and BAEs also remains difficult because of confounding factors such as concomitant medications, the underlying epilepsy itself, and psychosocial influences [17].

These cases highlight important clinical insights consistent with existing literature; behavioral toxicity may occur across all age groups and can manifest after initiating LEV [18]. Regular behavioral monitoring and early dose modification are recommended, especially in high-risk groups [19]. When significant psychiatric symptoms emerge, a multidisciplinary approach involving neurologists and psychiatrists is crucial. Discontinuation or substitution with alternative antiseizure medications such as valproate should be considered after carefully weighing the benefits of seizure control against potential behavioral risks. These BAEs were infrequently observed with LEV therapy [5-7]. Our study represents one of the few published case series from India, and to the best of our knowledge, it is the second such report from South India. Overall, the findings of this case series are consistent with previous limited evidence, indicating that, while LEV is an effective and generally well-tolerated antiepileptic agent, behavioral disturbances constitute a clinically relevant adverse effect that necessitates vigilance, prompt management, and individualized therapeutic decisions [20].

## Conclusions

This case series underscores the complex relationship between epilepsy, antiepileptic drug therapy, and neuropsychiatric manifestations. Behavioral disturbances observed in association with levetiracetam use emphasize the need for individualized pharmacotherapy. Most important is the integration of psychiatric evaluation into epilepsy management, particularly when patients exhibit new-onset behavioral or emotional symptoms. Continued prospective observational studies are needed to delineate the behavioral profiles of different antiepileptic drugs and to guide the evidence-based selection of therapy in patients who are vulnerable to psychiatric comorbidities.
